# Imbibition of Newtonian Fluids in Paper-like Materials with the Infinitesimal Control Volume Method

**DOI:** 10.3390/mi12111391

**Published:** 2021-11-12

**Authors:** Kui Song, Ruijie Huang, Xiaoling Hu

**Affiliations:** 1 College of Civil Engineering and Mechanics, Xiangtan University, Xiangtan 411105, China; ruijiehuang@126.com (R.H.); huxiaoling@xtu.edu.cn (X.H.); 2 Institute of Rheological Mechanics, Xiangtan University, Xiangtan 411105, China

**Keywords:** microfluidics, imbibition, paper-like material, paper-based microfluidic devices, infinitesimal control volume method

## Abstract

Paper-based microfluidic devices are widely used in point-of-care testing applications. Imbibition study of paper porous media is important for fluid controlling, and then significant to the applications of paper-based microfluidic devices. Here we propose an analytical approach based on the infinitesimal control volume method to study the imbibition of Newtonian fluids in commonly used paper-like materials. Three common paper shapes (rectangular paper strips, fan-shaped and circular paper sheets) are investigated with three modeling methods (corresponding to equivalent tiny pores with circle, square and regular triangle cross section respectively). A model is derived for liquid imbibition in rectangular paper strips, and the control equations for liquid imbibition in fan-shaped and circular paper sheets are also derived. The model is verified by imbibition experiments done using the mixed cellulose ester filter paper and pure water. The relation of imbibition distance and time is similar to that of the Lucas−Washburn (L−W) model. In addition, a new porosity measurement method based on the imbibition in circular paper sheets is proposed and verified. Finally, the flow rates are investigated. This study can provide guidance for the design of different shapes of paper, and for better applications of paper-based microfluidic devices.

## 1. Introduction

Paper has many advantages, such as a low cost, portability, compatibility with most biochemical reactions and high flexibility [1,2,3,4,5,6,7]; it can be combined with automation technologies to form programmable paper-based microfluidic devices for better control of fluidic sample transport, mixing and reaction, then to realize multiple biomarker detection, disease diagnosis, etc. [8,9,10,11,12,13,14,15]. Paper-based microfluidic devices have become a useful point-of-care testing (POCT) tool, however, there are some deficiencies, such as low sensitivity, poor reliability, and low reproducibility. Most devices can only provide yes/no tests and qualitative detection [16]. One important reason is the unclear underlying mechanism of liquid flow, imbibition, in paper porous media. Therefore, imbibition study is significant for the preparation of highly sensitive, multifunctional and stable paper-based microfluidic devices.

Various strategies have been proposed to adjust the imbibition flow rate. For instance, designing different paper shapes [17,18,19,20,21], designing hydrophobicity and a water barrier to realize space control of fluids [20,22], adjusting pore size and porosity of the paper [23], etc. Mathematical models have been developed to understand liquid imbibition behavior. A detailed review of various mathematical and numerical models on investigating the capillary-driven process in paper-like materials was presented by Liu et al. [24]. For instance, Darcy’s and Brinkman’s models can predict the velocity and pressure fields of liquid imbibition in porous materials [3,16]. However, Darcy’s law ignores the viscous effects and is a macroscopic law developed for saturated flow; it cannot solve time-dependent problems. Brinkman’s model adds the viscous term, but the dynamic viscosity can only be obtained by empirical methods. In addition, both Darcy’s and Brinkman’s models cannot obtain analytical solutions of imbibition for arbitrary geometric shapes [24,25].

Imbibition is self-driven using capillary forces without pumps, valves, or other external energy sources [26,27,28]. Therefore, it is usually modeled by the capillary bundle theory, which assumes that the pores are interconnected and homogeneously distributed, thus the porous medium acts like a bundle of identical capillary tubes with an effective tube radius [22,29,30]. If the inertial force and gravity are ignored, the relation of imbibition distance *x* and time *t* can be described by the Lucas−Washburn (L−W) model *x*~*t*^1/2^ [31]. The L−W model has been used to describe the overall behavior of liquid imbibition by fitting the effective capillary radius, and it assumes smooth and continuous cylindrical pores; but random porous media can hardly meet the conditions because of the complexity of pore structure. If the shape of a paper chip changes irregularly, the imbibition distance and the corresponding imbibition volume may not be linearly related. However, the use of effective radius does not consider this problem [6].

Actually, liquid imbibition depends on pore size, porosity, permeability, tortuosity, evaporation, etc. [31,32,33,34,35]. Various detailed models are developed to study the influence of the above factors on imbibition behaviors, for instance: considering boundary conditions, different sizes and shapes of pores, the tortuosity of imbibition streamlines in random porous media, the initial wetting-phase saturation, etc. [22,36,37]. Imbibition is also studied by appealing to the concerned ensemble-averaged transport with reference to the underlying molecular picture [38] or using fractal theory to describe the complex and random microstructure of porous media [39,40,41,42]. These models can provide reasonable predictions for liquid imbibition, but they need to introduce parameters related to imbibition tortuosity, pore shape, porosity, fractal dimensions, etc., and the parameters have to be determined by different experiments of porous materials.

The pore size of the paper ranges from 10^−3^ to 10^1^ microns [43], and we consider that if the pore length is infinitely small, each infinitely small segment pore can be seen as a small segment of capillary tube. Therefore, each infinitesimal control volume contains a large number of infinitely small segments of capillaries, then modeling study can be done based on the small segments of capillaries. For an isotropic porous medium with uniform pore size, the specifications of the infinitely small segments of capillaries can be considered as uniform ones. This infinitesimal control volume method is suitable for different paper shapes without considering the possible non-linear relationship between imbibition distance and the corresponding imbibition volume [6].

In this work, the imbibition of Newtonian fluids in paper-like materials is studied with theory and experiment. The paper-like materials are hydrophilic, planar, homogeneous and isotropic porous media. The evaporation effective is neglected for the short-time imbibition process [24]. Meanwhile, since the two-dimensional paper porous medium is very thin, we can assume that liquid only seeps inside the thin layer [44]. According to the momentum theorem, the change of total momentum *I* per unit time equals to the resultant force acting on the fluid. For a horizontally placed planar porous medium, the gravity can be neglected; the momentum equation is d*I*/d*t* = *F_σ_*−*F_μ_*, where *F_σ_* is capillary force, *F_μ_* is viscous force and *t* is time.

## 2. Materials and Methods

### 2.1. Imbibition Analysis with the Infinitesimal Control Volume Method

**Figure 1:**
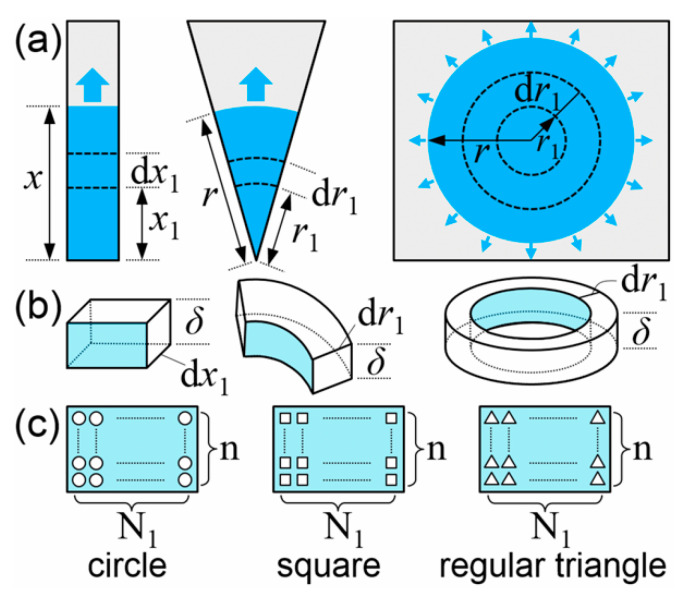
(**a**) Imbibition schematic of three shapes of paper: rectangular paper strips, fan-shaped paper sheets and circular paper sheets. (**b**) Schematic of the infinitesimal control volume. (**c**) A large number of small circles, squares or regular triangles on the cross section of the infinitesimal control volume, which corresponds to three modeling methods. *n* and *N*_1_ are the number of small circle layers in the thickness and width direction respectively.

We consider three common shapes of paper media, which include rectangular paper strips with different widths, fan-shaped paper sheets with different angles, and circular paper sheets (with an angle of 2*π*), as shown in Figure 1a. Similar to the application conditions of the L−W model [24], we assume that: (1) the evaporation and gravity force are ignored; (2) the boundary effect is not considered; (3) the material is hydrophilic, planar, homogeneous and isotropic porous medium (as mentioned above); (4) the imbibition liquid is considered to be laminar, incompressible.

The contents of the infinitesimal control volume method are: take a small segment as the infinitesimal control volume in the imbibition direction as shown in Figure 1b, where the length of the small segment for rectangular paper is d*x*, and the length for fan-shaped or circular paper sheets is d*r*; analyze the momentum and viscous force of the flow in the control volume; derive the total momentum and total viscous force by integration at a certain time *t*; list the governing equation combining with the capillary force; solve the equation to obtain the imbibition model last. For isotropic porous media with uniform pore size, we can use a large number of small circular, small square or small regular triangle cross-sectional pores of uniform size to represent the pores in the infinitesimal control volume. The volume of all the uniform small pores equals to the total volume of pores in the control volume. Because the length of the control volume is infinitely small, theoretical modeling can be described by encircling a large number of small circles, squares or regular triangles on a cross section of the control volume as shown in Figure 1c. A uniform small pore characterizes the area of one pore on the cross section, so the area of a small circle *S*_○_, a small square *S*_□_ and a small regular triangle *S*_△_ should equal to each other. Assume that the diameter of the small circle is *D*, the side lengths of the small square and regular triangle are *a* and *b* respectively, then,

(1) $$\pi {\left(\frac{D}{2}\right)}^{2}=a^{2}=\frac{\sqrt{3}}{4}b^{2}$$

For rectangular paper strips, assume that the thickness is *δ*, the width is *W*, and the porosity is *η*. When the imbibition length is *x* at a certain time *t*, the infinitesimal control volume at the *x*_1_ position is taken as shown in Figure 1a, where the length is d*x*_1_, the mass is d*m*_1_ = *ρδW*d*x*_1_*η*, and the velocity is *v*_1_ = *v*_1_(*x*_1_) = d*x*_1_/d*t*. Similarly, the mass of the control volume with d*x* length d*m* = *ρδW*d*xη*, and the velocity *v* = *v*(*x*) = d*x*/d*t*. Take the modeling method of the small circle cross-sectional pore as an example, assume that the pore volume of the infinitesimal control volume with d*x*_1_ length equals to the total volume of all *nN*_1_ small circular cross-sectional pores, where *n* and *N*_1_ are the number of small circle layers in the thickness and width direction respectively as shown in Figure 1c. Since the thickness is uniform, *n* is constant. Similarly, assume that the pore volume of the control volume with d*x* length equals to the total volume of all *nN* small circular cross-sectional pores. Then we have:

(2) $$\{\begin{array}{l} Wdx_{1}\delta \eta =nN_{1}\pi {\left(\frac{D}{2}\right)}^{2}dx_{1};\\ Wdx\delta \eta =nN\pi {\left(\frac{D}{2}\right)}^{2}dx. \end{array}$$

At a certain time, *t*, the imbibition flow rate *Q*_1_ at the *x*_1_ position equals to the flow rate *Q* at the *x* position according to the conservation of flow rate:

(3) $$Q_{1}=nN_{1}\pi {\left(\frac{D}{2}\right)}^{2}v_{1}=Q=nN\pi {\left(\frac{D}{2}\right)}^{2}v$$

The total momentum *I* of the flow in the segment with *x* length is:

(4) $$I=\int_{0}^{x}v_{1}dm_{1}=\int_{0}^{x}v_{1}\rho W\delta \eta dx_{1}$$

According to Equations (2)–(4), *I* is *ρWδηxv*. Assume that the contact angle is *θ*, the capillary force for one small circle pore is *F_σi_* = *πDσ*cos* θ* (*I* = 1, 2, …, *nN*), so the total capillary force is *F_σ_* = *nNF_σi_* = 4*Wδησ*cos* θ*/*D* combining with Equation (2). In the infinitesimal control volume with d*x*_1_ length, the flow resistance for one small circle pore is d*R_i_* = 128*μ*d*x*_1_/(*πD*^4^) (*I* = 1, 2, …, *nN*_1_) [45], then the total viscous force of the flow in the segment with *x* length can be derived using the parallel method, *F_μ_* = 32*Wμδηxv*/*D*^2^ (see the Supplementary Material). Assume that the initial length is *x*_0_ for rectangular paper, and *r*_0_ for fan-shaped or circular paper sheets. By solving the momentum equation (see the Supplementary Material), the relation of *x*-*t* is:

(5) $$x=\sqrt{2C_{1}\left[t+\frac{1}{C_{2}}\left(e^{-C_{2}t}-1\right)\right]+{x_{0}}^{2}}$$

The modeling methods of the small square and regular triangle cross-sectional pores are the same as that of the small circle cross-sectional pore, their corresponding coefficients *C*_1_ and *C*_2_ are given in Table 1 (see the Supplementary Material). The model (Equation (5)) shows that the imbibition distance is independent of the width of the paper strip, which is consistent with the research results of Elizalde et al. [6], Shou et al. [21] and Böhm et al. [46]. The imbibition velocity can be calculated by derivation:

(6) $$v=\frac{dx}{dt}=\frac{C_{1}\left(1-e^{-C_{2}t}\right)}{x}$$

Similarly, for the imbibition of fan-shaped or circular paper sheets, their governing equations of imbibition length *r* and time *t* can be derived, and they have the same form (see the Supplementary Material):

(7) $$\frac{d\left(r^{2}v\right)}{dt}=C_{3}r-C_{2}r^{2}v$$

where *C*_3_ is given in Table 1. The same equation indicates that the imbibition is independent of the angles of paper sheets. There is no analytical solution for Equation (7) and we can give the numerical result of it. In this study, we use the MATLAB software to do calculations and give the theoretical results (including the model result of Equation (5) and the numerical result of Equation (7)). The MATLAB codes are in the Supplementary Material.

### 2.2. Imbibition Experiment

**Figure 2:**
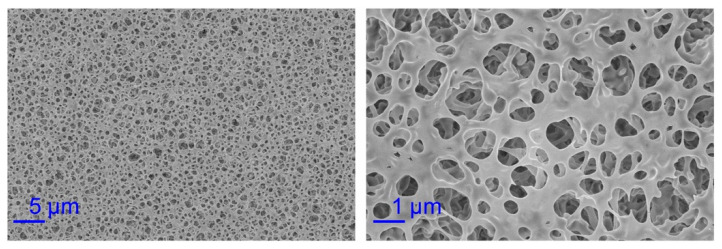
The scanning electron microscope (SEM) results of the mixed cellulose esters (MCE) paper surface. The left and right images have a magnification of 2000 and 10,000 times, respectively.

**Figure 3:**
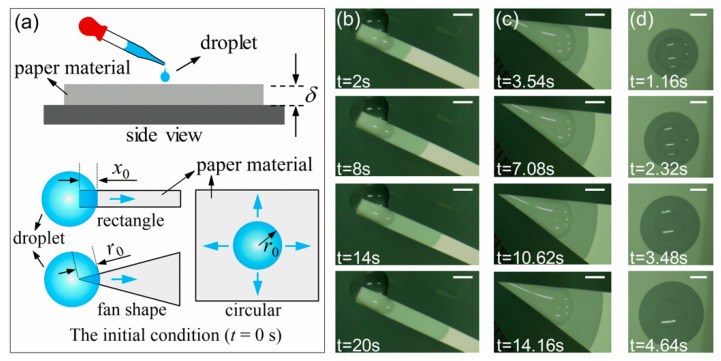
(**a**) Schematics of the experiment and the initial condition (*t* = 0 s). (**b**–**d**) shows the imbibition area of a rectangular paper strip, a fan-shaped paper sheet and a circular paper sheet at different times, respectively, where the scale bar is 2 mm.

In imbibition experiments, we chose mixed cellulose esters (MCE) filter paper as the planar porous medium. The pore size of the MCE paper is 0.45 μm, the porosity *η* is 79%, and the contact angle *θ* for water is 55.9° [47]. We used the scanning electron microscope (SEM, Zeiss Sigma 300, Carl Zeiss AG, Jena, Germany) to observe the microstructure of the MCE paper surface. The SEM results show that the pore size is uniform and consistent with the given result 0.45 μm as shown in Figure 2. Since *D* is the diameter of the small circle, so *D* equals to 0.45 μm. Here the surface tension *σ* is 0.0728 N/m, the thickness *δ* of an MCE paper is 0.15 mm, the dynamic viscosity μ of water is 1.005 × 10^−3^ kg/(ms) and the density *ρ* of water is 998.2 kg/m^3^. We closely stuck one side of the MCE paper with the transparent tape, so that the fluid dripped from one surface of the paper but did not leak from the other surface. In experiments, a pure water drop was dripped with a dropper onto the corresponding positions of the horizontally placed rectangular paper strips, fan-shaped or circular paper sheets, as shown in Figure 3a. Meanwhile, a CCD camera was used to capture the dynamic imbibition process. The schematic of the initial conditions (*t* = 0 s) including the initial length *x*_0_ for rectangular paper and *r*_0_ for fan-shaped or circular paper sheets are also shown in Figure 3a. Figure 3b–d shows the imbibition area of a rectangular paper strip, a fan-shaped and a circular paper sheet at different times, respectively. The results show that the imbibition area gradually increases with time, and an isotropic pore distribution ensures a circular imbibition front line [18]. In addition, we observed the imbibition areas for a long time in experiments at room temperature and found that the areas did not disappear for several minutes. Because the imbibition results were recorded in tens of seconds, the evaporation effect can be ignored. Data collection was done with data acquisition software used to obtain the experimental results of imbibition distance *x* (and *r*) as a function of time *t*.

## 3. Results and Discussion

**Figure 4:**
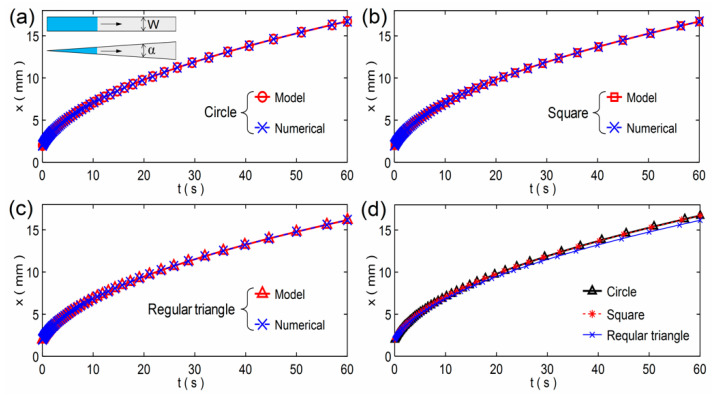
Comparisons of the model results and numerical solutions for the three modeling methods: (**a**) the small circle cross-sectional pore; (**b**) the small square cross-sectional pore; and (**c**) the small regular triangle cross-sectional pore. The illustration in (**a**) is a schematic of a rectangular paper strip with width *W* and a fan-shaped paper sheet with an angle *α*. (**d**) Comparisons of the model results for the three modeling methods. Without loss of generality, all the initial imbibition lengths are 2 mm.

Based on the results of the parameters, we can obtain the imbibition results according to the model Equation (5) or solve Equation (7) numerically. The model results and equation solutions were compared for the three paper shapes and the three modeling methods respectively, as shown in Figure 4. It is found that all the results are almost equal to each other when the initial imbibition lengths are the same (*x*_0_ = *r*_0_). According to the theoretical analysis, the imbibition result is independent of the paper width *W* and the angle *α*. That is, when the width and angle both tend to 0, the imbibition length is still the result obtained by the model and equation, respectively. As the illustration in Figure 4a shows that the two paper sheets will tend to two same lines when *W* and *α* both tend to 0, so the results of the model and equation are equal. As shown in Figure 4d, the model results are almost equal to each other for the three modeling methods, which means the three methods have little effect on the imbibition results.

**Figure 5:**
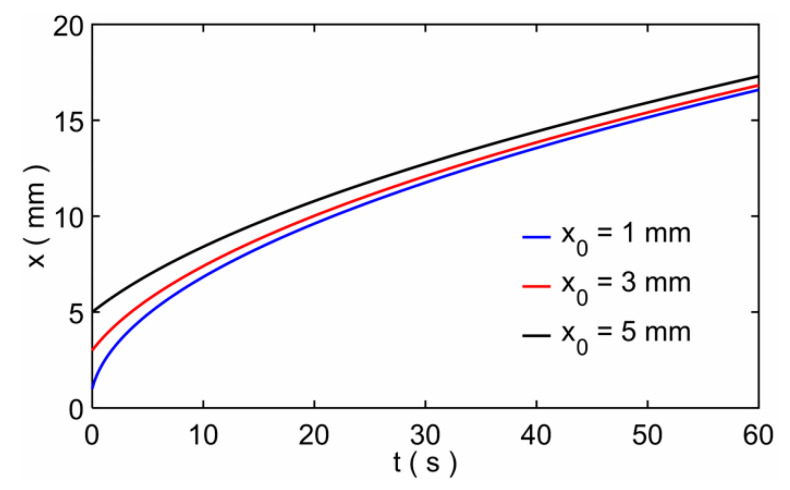
The results of imbibition distance *x* as a function of time *t* for different initial imbibition lengths.

**Figure 6:**
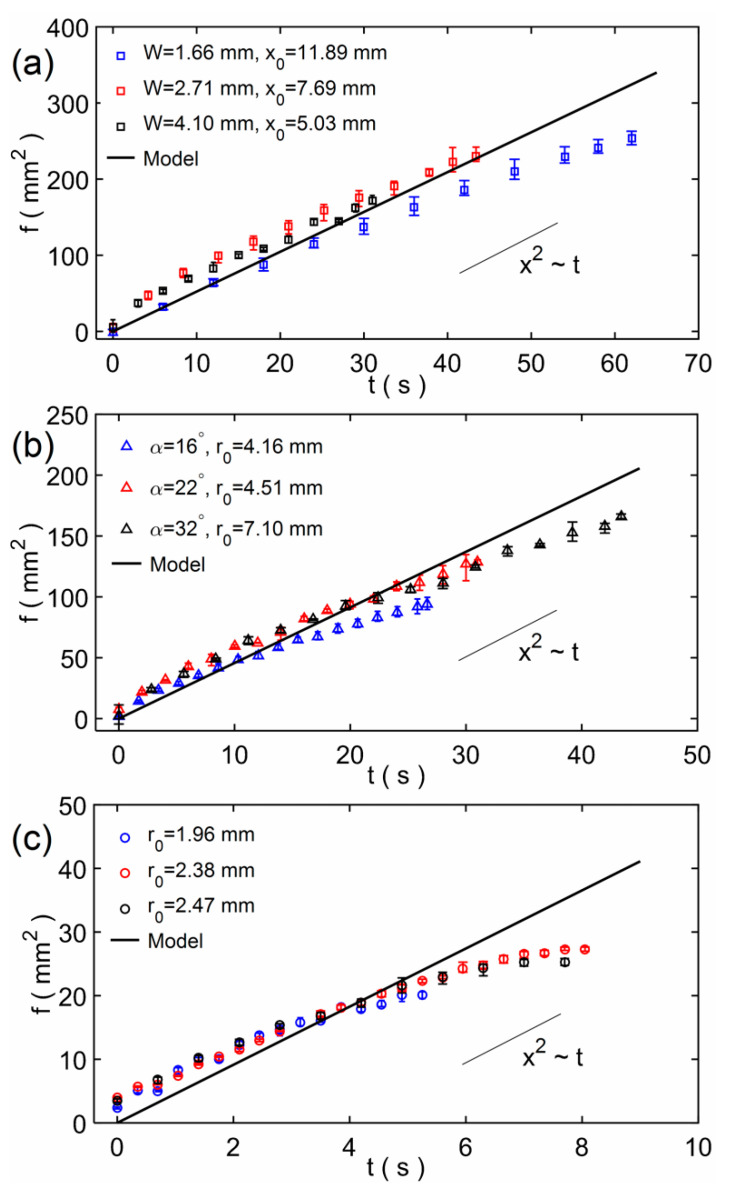
The *f*-*t* comparison results of the model and experiment: (**a**) the rectangular paper strip; (**b**) the fan-shaped paper sheet; and (**c**) the circular paper sheet. The marked *x*^2^~*t* rule shows that the relation of imbibition distance *x* and time *t* is similar to the L−W model.

According to the above analysis, the model result was used to compare with the experimental result. In addition, since the results of the three modeling methods are almost the same, we choose the model based on the modeling method of the small circle cross-sectional pore. Furthermore, as the model results show in Figure 5, the initial imbibition length has an effect on the imbibition results. In experiments, the initial imbibition length (*x*_0_ or *r*_0_) is difficult to control as a uniform one even for the same width rectangular paper strips (or the same angle fan-shaped paper sheets), and so it is with the mass of one droplet in each experiment. We changed the experimental data and the model here. Assume *f* = *x*^2^ − *x*_0_^2^, so *f* = 2*C*_1_ [*t* + (*e*^−*C*^^2*t*^ − 1)/*C*_2_] according to Equation (5), then the theoretical and experimental results of *f* were compared with each other. Figure 6a–c shows the *f*-*t* comparison results of the rectangular paper strip, the fan-shaped paper sheet and the circular paper sheet. In experiments, the rectangular paper strip has three different widths and the fan-shaped paper sheet has three different angles. The initial imbibition lengths are different for all the paper sheets; they are the average results of measurements. The model results agree with the experimental results, and the marked curve means the *x*^2^~*t* rule that shows the relation of imbibition distance *x* and time *t* is similar to the L−W model [32,48]. There are large errors when the imbibition time is very long. This is because we dripped one water drop every time in experiments, so the water gradually disappeared, and the imbibition distance *x* (or *r*) would not change in the end. For the theory model, water comes from an infinite reservoir and the imbibition distance increases for a very long time. So, the modeling method of this study is verified and can be applicable to different shapes of paper. When investigating the imbibition flow of a paper material, the imbibition results and flow rate can be predicted via this model by measuring the pore size and porosity of the material.

An important application of this study is the porosity measurement based on the imbibition in circular paper sheets. In experiments, a pure water drop was dripped onto a circular paper sheet. We can obtain the mass *m* of the water drop by measuring the total mass of the dropper before and after the dripping operation, and *m* is the difference between the two total masses. An electronic scale was used to measure droplet masses. The water drop volume is *V* = *m*/*ρ*. The water drop will fill up the pores of the imbibition area after enough time to form a stable circular area. The radius *R* of the circular area can be measured. Then for a circular paper area with thickness *δ* and porosity *η*, the total volume of the pores is *πR*^2^*δη*, we have *V* = *πR*^2^*δη*, so the porosity *η* is:

(8) $$\eta =\frac{V}{\pi R^{2}\delta }=\frac{m}{\pi \rho R^{2}\delta }$$

**Figure 7:**
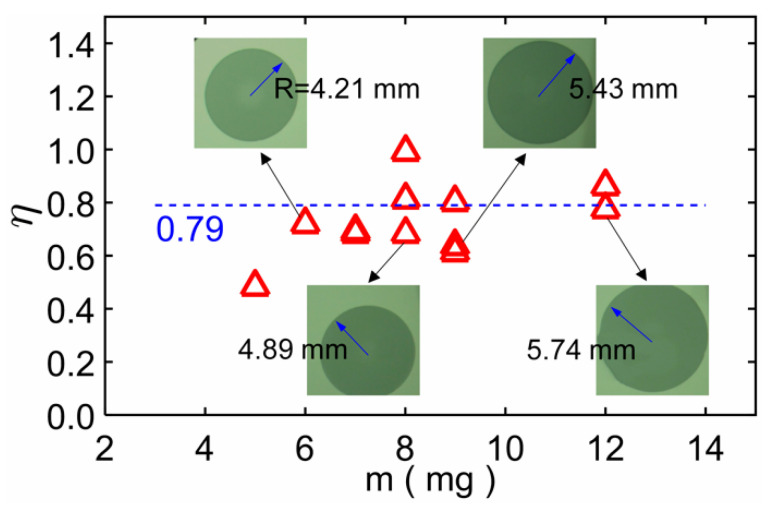
The results of porosity *η* as a function of mass *m*. Four results are marked with the corresponding experimental images. *η* changes around the given parameter 0.79 with *m*.

Since porosity is an inherent physical property of porous materials, the porosity *η* calculated via Equation (8) should not change with the mass *m*. We measured the mass of one droplet and the corresponding circular imbibition area and substituted the results into Equation (8) of the manuscript to obtain the porosity, and then compared the calculation result with the given porosity result 0.79. Figure 7 shows the results of *η* as a function of *m*, and four results are marked with the corresponding experimental images. The results show that the porosity *η* is independent of the drop mass *m*. As the sensitivity of the electronic scale (for measuring droplet mass) is 1 mg, and the measured droplet mass is only a few milligrams, this may have measurement errors. In addition, the measurement of imbibition area may have errors. So, the calculation result is quite different from the given porosity result 0.79, and the calculation result varies from 0.50 to 0.98, which is generally consistent with the given porosity 0.79.

**Figure 8:**
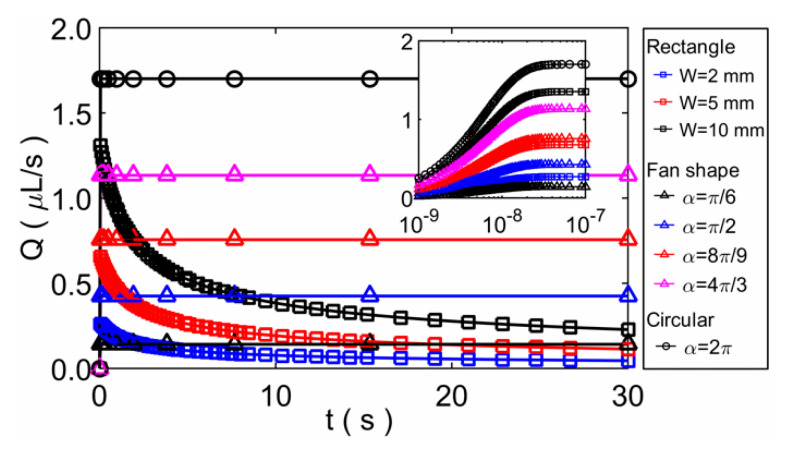
The results of volumetric flow rate *Q* as a function of time *t* for the three paper shapes.

In addition, the imbibition volumetric flow rate *Q* can be obtained based on the model and the known porosity. We found that the flow rates of the three modeling methods for a same paper shape have the same form, and the results are listed in Table 2 (see the Supplementary Material). Figure 8 shows the results of *Q* as a function of time *t* for the three paper shapes, where the rectangular paper strip includes three representative widths, the fan-shaped paper sheet includes four representative angles. The unit of *Q* is expressed with μL/s. The inset figure in Figure 8 shows the result of the volumetric flow rate *Q* during the very short period of time *t* at the beginning of the imbibition flow. It can be seen that *Q* increases rapidly at the beginning for all the paper sheets. Later, the flow rate for rectangular paper strip gradually decreases, and the flow rates for fan-shaped and circular paper sheets are almost unchanged.

## 4. Conclusions

In conclusion, we propose an analytical approach based on the infinitesimal control volume method to study the imbibition of Newtonian fluid in paper-like materials. Three common paper shapes (rectangular paper strips, fan-shaped and circular paper sheets) are investigated, and three modeling methods (about small circle, square or regular triangle cross-sectional pores) are used to derive the model specifically. We found that for one modeling method, the model results of the imbibition in rectangular paper strips and the numerical results of the governing equation for the imbibition in fan-shaped paper (or circular paper) sheets are equal for the same initial imbibition length. Meanwhile, the results of the three modeling methods are almost the same for one paper shape under the same conditions. The model is verified by the imbibition experiment, and the relation of imbibition distance and time is similar to that of the L−W model. In addition, a new porosity measurement method based on the imbibition in circular paper sheets is proposed and verified. The flow rates for the three paper shapes are investigated last. Additionally, this study can also provide guidance to the design of different shapes of paper, and then to control and obtain desired flow rates.

## 5. Patents

We have applied for a patent of the porosity measurement resulting from this work.

## Figures and Tables

**Table 1:** The results of coefficients *C*_1_, *C*_2_ and *C*_3_ for the three modeling methods.

Coefficient	Circle	Square	Regular Triangle
*C*_1_	$\frac{\sigma D\cos\theta }{8\mu }$	$\frac{\sigma a\cos\theta }{7.1\mu }$	$\frac{\sqrt{3}\sigma b\cos\theta }{20\mu }$
*C*_2_	$\frac{32\mu }{\rho D^{2}}$	$\frac{28.4\mu }{\rho a^{2}}$	$\frac{80\mu }{\rho b^{2}}$
*C*_3_	$\frac{4\sigma \cos\theta }{\rho D}$	$\frac{4\sigma \cos\theta }{\rho a}$	$\frac{4\sqrt{3}\sigma \cos\theta }{\rho b}$

**Table 2:** The volumetric flow rates *Q* for the three paper shapes.

Paper Shape	Rectangle	Fan Shape	Circular
*Q*	*Wδηv*	*αδη* *rv*	2*πδηrv*

## Data Availability

Not applicable.

## Supplementary Materials

The following are available online at https://www.mdpi.com/article/10.3390/mi12111391/s1.
