# Supplementary material for: Imbibition of Newtonian Fluids in Paper-like Materials with the Infinitesimal Control Volume Method
Source: Micromachines (Basel). 2021 Nov 12;12(11):1391. doi: 10.3390/mi12111391 (PMC8626007; doi:10.3390/mi12111391)
Supplement: Supplementary file 1 [file micromachines-12-01391-s001.zip › micromachines-1409401-supplementary.pdf]

micromachines

MDPI

Supplementary materials

Imbibition of Newtonian Fluids in Paper-Like Materials with the Infinitesimal Control Volume Method

Kui Song [1,2,*], Ruijie Huang [1] and Xiaoling Hu [1,2]

[1] College of Civil Engineering and Mechanics, Xiangtan University, Xiangtan 411105, China; ruijiehuang@126.com (R.H.); huxiaoling@xtu.edu.cn (X.H.)
[2] Institute of Rheological Mechanics, Xiangtan University, Xiangtan 411105, China;
* Correspondence: songkui@xtu.edu.cn (K.S.)

In theoretical analysis, according to the momentum theorem, the change of total momentum I per unit time in imbibition process equals to the resultant force acting on the fluid. For a horizontally placed porous medium, the gravity can be neglected, and the momentum equation is,

$$\frac{\mathrm{d}I}{\mathrm{d}t} = F_\sigma - F_\mu \tag{S1}$$

Copyright: © 2021 by the authors. Licensee MDPI, Basel, Switzerland. This article is an open access article distributed under the terms and conditions of the Creative Commons Attribution (CC BY) license (https://creativecommons.org/licenses/by/4.0/).

where F_σ is the capillary force, F_μ is the viscous force and t is time.

The imbibition of hydrophilic Newtonian fluid in three common paper shapes are investigated. The three paper shapes are rectangular paper strips, fan-shaped paper sheets with different angles and circular paper sheets as shown in Figure 1(a) of the paper, and all of them are the homogeneous, isotropic and planar porous media.

1. The imbibition of hydrophilic Newtonian fluid in rectangular paper strips

For rectangular paper strips, assume that the thickness of the paper strip is δ, the width is W, and the porosity is η. For a imbibition length x at a certain time t, the infinitesimal control volume at the x_1 position is investigated as shown in Figure 1(b) of the paper, where the length of the control volume is $\mathrm{d}x_1$. The mass of the control volume is,

$$\mathrm{d}m_1 = \rho W \delta \eta \mathrm{d}x_1 \tag{S2}$$

The corresponding velocities at the x_1 and x positions are,

$$\begin{cases} v_1 = v_1(x_1) = \dfrac{\mathrm{d}x_1}{\mathrm{d}t}, \\ v = v(x) = \dfrac{\mathrm{d}x}{\mathrm{d}t}. \end{cases} \tag{S3}$$

Take the modeling method of the small circle cross-sectional pore as an example, assume that the pore volume of the infinitesimal control volume with $\mathrm{d}x_1$ length is equal to the total volume of all nN_1 small circular cross-sectional pores, where n is the number of small circle layers in the thickness direction, and N_1 is the number of small circle layers in the width direction, as shown in Figure 1(c) of the paper. Since the thickness of the paper medium is uniform, so n is constant (for rectangular paper strips, N_1 is also constant

because of the constant width, but for fan-shaped and circular paper sheets, N_1 is variable). Similarly, assume that the pore volume of the infinitesimal control volume with $\mathrm{d}x$ length is equal to the total volume of all *nN* small circular cross-sectional pores. Then we have,

$$\begin{cases} W\mathrm{d}x_1\delta\eta = nN_1\pi(\frac{D}{2})^2\,\mathrm{d}x_1; \\ W\mathrm{d}x\delta\eta = nN\pi(\frac{D}{2})^2\,\mathrm{d}x. \end{cases} \tag{S4}$$

At a certain time *t*, the volumetric imbibition flow rate Q_1 at the x_1 position equals to the volumetric flow rate *Q* at the *x* position according to the conservation of flow rate,

$$Q_1 = nN_1\pi(\frac{D}{2})^2 v_1 = Q = nN\pi(\frac{D}{2})^2 v \tag{S5}$$

The total momentum *I* of the flow in the segment with *x* length is,

$$I = \int_0^x v_1\mathrm{d}m_1 = \int_0^x v_1\rho W\delta\eta\mathrm{d}x_1 \tag{S6}$$

According to Equations (S2), (S3) and (S6), the total momentum *I* is $\rho W\delta\eta xv$. Assume that the contact angle is θ, the capillary force for one small circle cross section pore is,

$$F_{\sigma i} = \pi D\sigma\cos\theta, (i = 1, 2, \cdots, nN) \tag{S7}$$

Combine with Equation (S4), the total capillary force is,

$$F_\sigma = nNF_{\sigma i} = \frac{4W\delta\eta\sigma\cos\theta}{D} \tag{S8}$$

The total viscous force of the flow in the segment with *x* length can be derived using the parallel method. In the segment of $\mathrm{d}x_1$ length, the flow resistance for one small circle cross section pore is,

$$\mathrm{d}R_j = \frac{128\mu}{\pi D^4}\mathrm{d}x_1, (j = 1, 2, \cdots, nN_1) \tag{S9}$$

According to the parallel method, we have,

$$\frac{1}{\mathrm{d}R_{\mathrm{hydk}}} = \frac{1}{\mathrm{d}R_1} + \frac{1}{\mathrm{d}R_2} + \cdots + \frac{1}{\mathrm{d}R_{\mathrm{N_1}}} = \frac{\pi D^4 N_1}{128\mu\mathrm{d}x_1}, (k = 1, 2, \cdots, n) \tag{S10}$$

So,

$$\mathrm{d}R_{\mathrm{hydk}} = \frac{128\mu\mathrm{d}x_1}{\pi D^4 N_1}, (k = 1, 2, \cdots, n) \tag{S11}$$

Again, according to the parallel method,

$$\frac{1}{\mathrm{d}R_{\mathrm{hyd}}} = \frac{1}{\mathrm{d}R_{\mathrm{hyd1}}} + \frac{1}{\mathrm{d}R_{\mathrm{hyd2}}} + \cdots + \frac{1}{\mathrm{d}R_{\mathrm{hydn}}} = \frac{\pi D^4 nN_1}{128\mu\mathrm{d}x_1} \tag{S12}$$

So, the flow resistance $\mathrm{d}R_{\mathrm{hyd}}$ for the infinitesimal control volume with $\mathrm{d}x_1$ length is,

$$\mathrm{d}R_{\mathrm{hyd}} = \frac{128\mu \mathrm{d}x_1}{\pi D^4 n N_1} \tag{S13}$$

Based on Equations (S5) and (S13), the pressure drop $\mathrm{d}p_1$ for the infinitesimal control volume with $\mathrm{d}x_1$ length is,

$$\mathrm{d}p_1 = \mathrm{d}R_{\mathrm{hyd}} Q_1 = \frac{32\mu v \mathrm{d}x_1}{D^2} \tag{S14}$$

Then the viscous force for the infinitesimal control volume is,

$$\mathrm{d}F_{\mu 1} = \mathrm{d}p_1 n N_1 \pi (\frac{D}{2})^2 = 8\pi\mu n N v \mathrm{d}x_1 \tag{S15}$$

So, combine with Equation (S4), the total viscous force F_μ of the flow in the segment with *x* length is,

$$F_\mu = \int_0^x \mathrm{d}F_{\mu 1} = \frac{32W\mu\delta\eta x v}{D^2} \tag{S16}$$

Finally, the momentum equation becomes,

$$\frac{\mathrm{d}(\rho W\delta\eta x v)}{\mathrm{d}t} = \frac{4W\delta\eta\sigma\cos\theta}{D} - \frac{32W\mu\delta\eta x v}{D^2} \tag{S17}$$

The initial condition of Equation (S17) is, $t = 0,\ x = x_0$, and its solution is,

$$x = \sqrt{2C_1[t + \frac{1}{C_2}(e^{-C_2 t} - 1)] + x_0{}^2} \tag{S18}$$

where $C_1 = \frac{\sigma D\cos\theta}{8\mu},\ C_2 = \frac{32\mu}{\rho D^2}$.

The imbibition velocity can be got by derivation:

$$v = \frac{\mathrm{d}x}{\mathrm{d}t} = \frac{C_1(1 - e^{-C_2 t})}{x} \tag{S19}$$

The volumetric flow rate *Q* is,

$$Q = nN\pi(\frac{D}{2})^2 v = W\delta\eta v \tag{S20}$$

2. The imbibition of hydrophilic Newtonian fluid in fan-shaped paper sheets

For fan-shaped paper sheets, assume that the thickness of the paper sheet is *δ*, the angle is *α* (in radians), and the porosity is *η*. When the imbibition length is *r* at a certain time *t*, the infinitesimal control volume at the r_1 position is considered as shown in Figures 1(a) and 1(b) of the paper, where the length of the control volume is $\mathrm{d}r_1$. The mass of the control volume is,

$$\mathrm{d}m_1 = \rho\delta\eta\alpha r_1 \mathrm{d}r_1 \tag{S21}$$

The corresponding velocities at the r_1 and *r* positions are,

$$\begin{cases} v_1 = v_1(r_1) = \dfrac{\mathrm{d}r_1}{\mathrm{d}t}, \\ v = v(r) = \dfrac{\mathrm{d}r}{\mathrm{d}t}. \end{cases} \tag{S22}$$

Take the modeling method of the small circle cross-sectional pore as an example, assume that the pore volume of the infinitesimal control volume with $\mathrm{d}r_1$ length is equal to the total volume of all nN_1 small circular cross-sectional pores, where n is the number of small circle layers in the thickness direction, and N_1 is the number of small circle layers in the width direction, as shown in Figure S1(c). Similarly, assume that the pore volume of the infinitesimal control volume with $\mathrm{d}r$ length is equal to the total volume of all nN small circular cross-sectional pores. Then we have,

$$\begin{cases} \delta\eta\alpha r_1 \mathrm{d}r_1 = nN_1\pi(\dfrac{D}{2})^2 \mathrm{d}r_1; \\ \delta\eta\alpha r \mathrm{d}r = nN\pi(\dfrac{D}{2})^2 \mathrm{d}r. \end{cases} \tag{S23}$$

At a certain time *t*, the imbibition volumetric flow rate Q_1 at the r_1 position equals to the volumetric flow rate *Q* at the *r* position according to the conservation of flow rate,

$$Q_1 = nN_1\pi(\frac{D}{2})^2 v_1 = Q = nN\pi(\frac{D}{2})^2 v \tag{S24}$$

The total momentum *I* of the flow in the segment with radius *r* is,

$$I = \int_0^r v_1 \mathrm{d}m_1 = \int_0^r v_1 \rho\delta\eta\alpha r_1 \mathrm{d}r_1 \tag{S25}$$

According to Equations (S21), (S23), (S24) and (S25), the total momentum *I* is $\rho\delta\eta\alpha r^2 v$. Assume that the contact angle is θ, the capillary force for one small circle cross section pore is,

$$F_{\sigma i} = \pi D\sigma\cos\theta, (i = 1, 2, \cdots, nN) \tag{S26}$$

Combine with Equation (S23), the total capillary force is,

$$F_\sigma = nNF_{\sigma i} = \frac{4\delta\eta\sigma\alpha r\cos\theta}{D} \tag{S27}$$

The total viscous force of the flow in the segment with *r* length can be derived using the parallel method. In the segment of $\mathrm{d}r_1$ length, the flow resistance for one small circle cross section pore is,

$$\mathrm{d}R_j = \frac{128\mu}{\pi D^4}\mathrm{d}r_1, (j = 1, 2, \cdots, nN_1) \tag{S28}$$

According to the parallel method, we have,

$$\frac{1}{\mathrm{d}R_{\mathrm{hydk}}} = \frac{1}{\mathrm{d}R_1} + \frac{1}{\mathrm{d}R_2} + \cdots + \frac{1}{\mathrm{d}R_{\mathrm{N}_1}} = \frac{\pi D^4 N_1}{128\mu \mathrm{d}r_1}, (k = 1, 2, \cdots, n) \tag{S29}$$

So,

$$\mathrm{d}R_{\mathrm{hydk}} = \frac{128\mu \mathrm{d}r_1}{\pi D^4 N_1}, (k = 1, 2, \cdots, n) \tag{S30}$$

Again, according to the parallel method,

$$\frac{1}{\mathrm{d}R_{\mathrm{hyd}}}=\frac{1}{\mathrm{d}R_{\mathrm{hyd1}}}+\frac{1}{\mathrm{d}R_{\mathrm{hyd2}}}+\cdots+\frac{1}{\mathrm{d}R_{\mathrm{hydn}}}=\frac{\pi D^4 nN_1}{128\mu \mathrm{d}r_1} \quad \text{(S31)}$$

So, the flow resistance $\mathrm{d}R_{\mathrm{hyd}}$ for the infinitesimal control volume with $\mathrm{d}r_1$ length is,

$$\mathrm{d}R_{\mathrm{hyd}}=\frac{128\mu \mathrm{d}r_1}{\pi D^4 nN_1} \quad \text{(S32)}$$

Based on Equations (S24) and (S32), the pressure drop $\mathrm{d}p_1$ for the infinitesimal control volume with $\mathrm{d}r_1$ length is,

$$\mathrm{d}p_1=\mathrm{d}R_{\mathrm{hyd}}Q_1=\frac{32\mu v_1 \mathrm{d}r_1}{D^2} \quad \text{(S33)}$$

Then the viscous force for the infinitesimal control volume is,

$$\mathrm{d}F_{\mu 1}=\mathrm{d}p_1 nN_1\pi(\frac{D}{2})^2=8\pi\mu nNv\mathrm{d}r_1 \quad \text{(S34)}$$

So, combine with Equation (S23), The total viscous force F_μ of the flow in the segment with radius *r* is,

$$F_\mu=\int_0^r \mathrm{d}F_{\mu 1}=\frac{32\mu\delta\eta\alpha r^2 v}{D^2} \quad \text{(S35)}$$

Finally, the momentum equation becomes,

$$\frac{\mathrm{d}(\rho\delta\eta\alpha r^2 v)}{\mathrm{d}t}=\frac{4\delta\eta\sigma\alpha r\cos\theta}{D}-\frac{32\mu\delta\eta\alpha r^2 v}{D^2} \quad \text{(S36)}$$

The initial condition of Equation (S36) is, $t=0, r=r_0$, and it can be simplified as,

$$\frac{\mathrm{d}(r^2 v)}{\mathrm{d}t}=C_3 r-C_2 r^2 v \quad \text{(S37)}$$

where $C_2=\frac{32\mu}{\rho D^2}, C_3=\frac{4\sigma\cos\theta}{\rho D}$.

The volumetric flow rate *Q* is,

$$Q=nN\pi(\frac{D}{2})^2 v=\alpha\delta\eta r v \quad \text{(S38)}$$

3. The imbibition of hydrophilic Newtonian fluid in circular paper sheets

For circular paper sheets, it is the situation of the fan-shaped paper sheets where the angle is 2π. Assume that the thickness of the paper sheet is *δ* and the porosity is *η*. When the imbibition length is *r* at a certain time *t*, the infinitesimal control volume at the r_1 position is considered as shown in Figures 1(a) and 1(b) of the paper, where the length of the control volume is $\mathrm{d}r_1$. The mass of the control volume is,

$$\mathrm{d}m_1=2\pi\rho\delta\eta r_1 \mathrm{d}r_1 \quad \text{(S39)}$$

The corresponding velocities at the r_1 and r positions are,

$$\begin{cases} v_1 = v_1(r_1) = \dfrac{\mathrm{d}r_1}{\mathrm{d}t}, \\ v = v(r) = \dfrac{\mathrm{d}r}{\mathrm{d}t}. \end{cases} \tag{S40}$$

Take the modeling method of the small circle cross-sectional pore as an example, assume that the pore volume of the infinitesimal control volume with $\mathrm{d}r_1$ length is equal to the total volume of all nN_1 small circular cross-sectional pores, where n is the number of small circle layers in the thickness direction, and N_1 is the number of small circle layers in the width direction, as shown in Figure 1(c) of the paper. Similarly, assume that the pore volume of the infinitesimal control volume with $\mathrm{d}r$ length is equal to the total volume of all nN small circular cross-sectional pores. Then we have,

$$\begin{cases} 2\pi\delta\eta r_1 \mathrm{d}r_1 = nN_1\pi(\dfrac{D}{2})^2 \mathrm{d}r_1; \\ 2\pi\delta\eta r \mathrm{d}r = nN\pi(\dfrac{D}{2})^2 \mathrm{d}r. \end{cases} \tag{S41}$$

At a certain time t, the imbibition flow rate Q_1 at the r_1 position equals to the volumetric flow rate Q at the r position according to the conservation of flow rate,

$$Q_1 = nN_1\pi(\frac{D}{2})^2 v_1 = Q = nN\pi(\frac{D}{2})^2 v \tag{S42}$$

The total momentum I of the flow in the segment with radius r is,

$$I = \int_0^r v_1 \mathrm{d}m_1 = \int_0^r v_1 2\pi\rho\delta\eta r_1 \mathrm{d}r_1 \tag{S43}$$

According to Equations (S39), (S41), (S42) and (S43), the total momentum I is $2\pi\rho\delta\eta r^2 v$. Assume that the contact angle is θ, the capillary force for one small circle cross section pore is,

$$F_{\sigma i} = \pi D\sigma\cos\theta, (i = 1, 2, \cdots, nN) \tag{S44}$$

Combine with Equation (S41), the total capillary force is,

$$F_\sigma = nNF_{\sigma i} = \frac{8\pi\sigma\delta\eta r\cos\theta}{D} \tag{S45}$$

The total viscous force of the flow in the segment with r length can be derived using the parallel method. In the segment of $\mathrm{d}r_1$ length, the flow resistance for one small circle cross section pore is,

$$\mathrm{d}R_j = \frac{128\mu}{\pi D^4}\mathrm{d}r_1, (j = 1, 2, \cdots, nN_1) \tag{S46}$$

According to the parallel method, we have,

$$\frac{1}{\mathrm{d}R_{\mathrm{hyd}k}} = \frac{1}{\mathrm{d}R_1} + \frac{1}{\mathrm{d}R_2} + \cdots + \frac{1}{\mathrm{d}R_{\mathrm{N}_1}} = \frac{\pi D^4 N_1}{128\mu \mathrm{d}r_1}, (k = 1, 2, \cdots, n) \tag{S47}$$

So,

$$\mathrm{d}R_{\mathrm{hydk}}=\frac{128\mu\mathrm{d}r_1}{\pi D^4N_1},(k=1,2,\cdots,n) \quad (S48)$$

Again, according to the parallel method,

$$\frac{1}{\mathrm{d}R_{\mathrm{hyd}}}=\frac{1}{\mathrm{d}R_{\mathrm{hyd1}}}+\frac{1}{\mathrm{d}R_{\mathrm{hyd2}}}+\cdots+\frac{1}{\mathrm{d}R_{\mathrm{hydn}}}=\frac{\pi D^4nN_1}{128\mu\mathrm{d}r_1} \quad (S49)$$

So, the flow resistance $\mathrm{d}R_{\mathrm{hyd}}$ for the infinitesimal control volume with $\mathrm{d}r_1$ length is,

$$\mathrm{d}R_{\mathrm{hyd}}=\frac{128\mu\mathrm{d}r_1}{\pi D^4nN_1} \quad (S50)$$

Based on Equations (S42) and (S50), the pressure drop $\mathrm{d}p_1$ for the infinitesimal control volume with $\mathrm{d}r_1$ length is,

$$\mathrm{d}p_1=\mathrm{d}R_{\mathrm{hyd}}Q_1=\frac{32\mu v_1\mathrm{d}r_1}{D^2} \quad (S51)$$

Then the viscous force for the infinitesimal control volume is,

$$\mathrm{d}F_{\mu1}=\mathrm{d}p_1nN_1\pi(\frac{D}{2})^2=8\pi\mu nNv\mathrm{d}r_1 \quad (S52)$$

So, combine with Equation (S41), The total viscous force F_μ of the flow in the segment with radius *r* is,

$$F_\mu=\int_0^r\mathrm{d}F_{\mu1}=\frac{64\pi\mu\delta\eta r^2v}{D^2} \quad (S53)$$

Finally, the momentum equation becomes,

$$\frac{\mathrm{d}(2\pi\rho\delta\eta r^2v)}{\mathrm{d}t}=\frac{8\pi\sigma\delta\eta r\cos\theta}{D}-\frac{64\pi\mu\delta\eta r^2v}{D^2} \quad (S54)$$

The initial condition of Equation (S54) is, $t=0, r=r_0$, and it can be simplified as,

$$\frac{\mathrm{d}(r^2v)}{\mathrm{d}t}=C_3r-C_2r^2v \quad (S55)$$

Equation (S55) is the same as Equation (S37) of the fluid imbibition in fan-shaped paper sheets, where $C_2=\frac{32\mu}{\rho D^2}, C_3=\frac{4\sigma\cos\theta}{\rho D}$.

The volumetric flow rate *Q* is,

$$Q=nN\pi(\frac{D}{2})^2v=2\pi\delta\eta rv \quad (S56)$$

4. The modeling methods of the small square and small regular triangle cross-sectional pores, and the corresponding flow rates

For the modeling methods of the small square and small regular triangle cross-sectional pores, it is also assumed that the pore volume of the infinitesimal control volume

with $\mathrm{d}r_1$ length is equal to the total volume of all nN_1 small square (or small regular triangle) cross-sectional pores, where n is the number of small square (or small regular triangle) layers in the thickness direction, and N_1 is the number of small square (or small regular triangle) layers in the width direction, as shown in Figure 1(c) of the paper. Similarly, assume that the pore volume of the infinitesimal control volume with $\mathrm{d}r$ length is equal to the total volume of all nN small square (or small regular triangle) cross-sectional pores.

For the isotropic porous medium with uniform pore size, we can use a large number of small circular, small square or small regular triangle cross-sectional pores of uniform size to represent the pores in the infinitesimal control volume. Then the volume of all the uniform small pores equals to the volume of pores in the infinitesimal control volume. Because the length of the control volume is infinitely small, theoretical modeling can be described by encircling a large number of small circles, small squares or small regular triangles on the corresponding cross section of the infinitesimal control volume, and the small circle, small square and small regular triangle characterize the area of one pore on the cross section as shown in Figure 1(c) of the paper. Assume that the diameter of the small circle is D, the side length of the small square is a, and the side length of the small regular triangle is b, then the area of a small circle $S_{\circ}$, a small square $S_{\square}$ and a small regular triangle $S_{\triangle}$ should equal to each other, i.e.,

$$S_{\circ} = S_{\square} = S_{\triangle} \quad \text{(S57)} \quad (6)$$

or,

$$\pi(\frac{D}{2})^2 = a^2 = \frac{\sqrt{3}}{4}b^2 \quad \text{(S58)}$$

The modeling methods of the small square and small regular triangle cross-sectional pores are similar to that of the small circle cross-sectional pore, and their corresponding coefficients C_1, C_2 and C_3 are given in Table 1 of the paper.

As shown in Figure 1(c), for a same paper shape, the total number of the small circles $(nN_1)_{\circ}$, small squares $(nN_1)_{\square}$ and small regular triangles $(nN_1)_{\triangle}$ on the corresponding cross section are equal, i.e.,

$$(nN_1)_{\circ} = (nN_1)_{\square} = (nN_1)_{\triangle} \quad \text{(S59)}$$

Based on Equations (S57) and (S59), the total flow rate of the three modeling methods ($Q_{\circ}$ for small circles, $Q_{\square}$ for small squares and $Q_{\triangle}$ for small regular triangles) equals to each other, i.e.,

$$(nN_1)_{\circ}S_{\circ}v = (nN_1)_{\square}S_{\square}v = (nN_1)_{\triangle}S_{\triangle}v \quad \text{(S60)}$$

or,

$$Q_{\circ} = Q_{\square} = Q_{\triangle} \quad \text{(S61)}$$

So, the volumetric flow rates of the three modeling methods for a same paper shape have the same expressions, their results are listed in Table 2 of the paper.

5. The Matlab codes for the calculation of the theoretical results

```
clear all; clc;
delta=0.15*10^(-3);
rho=998.2;
```

```
D=0.45*10^(-6);
eta=0.79;
sigma=0.0728;
mu=0.001005;
theta=55.9*pi/180;
w1=D; w2=D*sqrt(pi)/2; w3=D*sqrt(pi)/3^0.25;
C1=sigma*w1*cos(theta)/(8*mu);
C2=32*mu/(rho*w1^2);
C3=sigma*w2*cos(theta)/(7.1*mu);
C4=28.4*mu/(rho*w2^2);
C5=3^0.5*sigma*w3*cos(theta)/(20*mu);
C6=80*mu/(rho*w3^2);
x0=0.002;

%% The model result
x=(2*C1*(t+exp(-C2*t)/C2-1/C2)+x0^2)^0.5;
f=2*C1*(t+exp(-C2*t)/C2-1/C2);

%% The numerical result
% main file
[t,r]=ode23s('sss',[0,60],[0.002 0],[],delta,w1,sigma,theta,rho,mu);
k=length(t);
n1=1:1:k;
t=t(n1);
r_1=r(n1,1);
r_2=r(n1,2);

% function file
function du=sss(t,u,dummy,delta,w,sigma,theta,rho,mu)
C1=4*sigma*cos(theta)/(rho*w); C2=32*mu/(rho*w^2); % small circle
C1=4*sigma*cos(theta)/(rho*w); C2=28.4*mu/(rho*w^2); % small square
C1=4*sqrt(3)*sigma*cos(theta)/(rho*w); C2=80*mu/(rho*w^2); % small triangle
du=[u(2); (C1*u(1)-C2*u(1)^2*u(2)-2*u(1)*u(2)^2)/u(1)^2];
end
```
